# Community Violence Exposure and Eating Disorder Symptoms among Belgian, Russian and US Adolescents: Cross-Country and Gender Perspectives

**DOI:** 10.1007/s10578-023-01590-1

**Published:** 2023-08-22

**Authors:** Johan Isaksson, Martina Isaksson, Andrew Stickley, Robert Vermeiren, Roman Koposov, Mary Schwab-Stone, Vladislav Ruchkin

**Affiliations:** 1 Child and Adolescent Psychiatry Unit, Department of Medical Sciences, Uppsala University, Uppsala, Sweden; 2 Center of Neurodevelopmental Disorders (KIND), Centre for Psychiatry Research, Department of Women’s and Children’s Health, Karolinska Institutet & Stockholm Health Care Services, Region Stockholm, Sweden; 3 Department of Preventive Intervention for Psychiatric Disorders, National Institute of Mental Health, National Center of Neurology and Psychiatry, Tokyo, Japan; 4 Stockholm Center for Health and Social Change (SCOHOST), Södertörn University, Huddinge, Sweden; 5 Department of Child and Adolescent Psychiatry Curium, Leiden University Medical Center, Leiden, Netherlands; 6 Regional Centre for Child and Youth Mental Health and Child Welfare, UiT The Arctic University of Norway, Tromsö, Norway; 7 Child Study Center, Yale University School of Medicine, New Haven, USA; 8 Sala Forensic Psychiatric Clinic, Sala, Sweden; 9 Department of Epidemiology and Modern Technologies of Vaccination, Institute of Professional Education, Sechenov First Moscow State Medical University, Moscow, Russia

**Keywords:** Anorexia, Bulimia nervosa, Eating disorder, Cross-culture, Trauma, Gender, Community violence, PTSD

## Abstract

Community violence exposure (CVE) is one of the most common adverse childhood experiences worldwide. Despite this, its potential effect on disordered eating in adolescents from different cultures is underexplored. In the present cross-sectional study, self-reported data were collected from 9751 students (Mean age = 14.27) from Belgium, Russia and the US on CVE (witnessing violence and violence victimization), eating disorder (ED) symptoms (ED thoughts with associated compensatory behaviors), and comorbid symptoms of posttraumatic stress, depression and anxiety. Increased CVE (from no exposure to witnessing to victimization) was associated with more ED symptoms, and the associations remained significant after adjusting for comorbid conditions. The associations were similar for adolescents across the three countries. No gender differences were observed in the association between CVE and ED symptoms, even though girls in general reported more ED symptoms than boys. We conclude that CVE appears to be associated with ED symptoms in three culturally different samples of adolescents.

**Supplementary Information:**

The online version contains supplementary material available at 10.1007/s10578-023-01590-1.

## Introduction

Eating disorder (ED) symptoms include fear of gaining weight, excessive concern with body shape and weight, and an overpowering desire to be thin, leading to the use of compensatory behaviors aimed at weight loss [1]. Previous research has indicated that ED symptoms can greatly impair physical health, quality of life and daily functioning [2]. Although the prevalence of ED symptoms tends to be much higher among females [3], this gender difference is less pronounced in adolescence, with the prevalence of anorexia nervosa, bulimia nervosa, and binge eating disorder ranging from 0.3 to 2.3% in adolescent females and from 0.3 to 1.3% in adolescent males [4]. While ED symptoms often occur during late adolescence, recent research has indicated that the age of their onset is decreasing and that their prevalence may be increasing at a faster rate among males [5].

The etiology of EDs is multi-factorial. In particular, although heritability is considered as a significant factor, explaining just over half of the variance in their occurrence [6, 7], a considerable part of the variance is also explained by environmental factors. One of the factors that is highly relevant in this context is exposure to traumatic experiences, where diverse types of interpersonal trauma, including emotional, physical, and sexual abuse, have been associated with a greater risk for all types of EDs in adolescents [8]. Similarly, posttraumatic stress disorder (PTSD), resulting from a traumatic exposure, is common among individuals with EDs [9], with more than one-third of adults with bulimia nervosa and one-fourth of those with binge eating disorder having a lifetime history of PTSD [10]. In a recent review, the prevalence of comorbid PTSD in patients with EDs ranged between 9% and 24% [11]. Although, the mechanisms underlying the relation between traumatic experiences and EDs have yet to be determined, it has been suggested that trauma-related factors such as self-criticism, low self-worth, self-punishment, and negative cognitions and affect, may be particularly relevant for ED behaviors, including controlling weight and shape [10].

While previous research has focused extensively on the associations between various types of trauma and EDs, until now, the association between community violence exposure (CVE) and EDs has been understudied. This may be an important oversight, as CVE is one of the most common adverse experiences in childhood [12] and has been associated with a variety of detrimental outcomes. For example, one form of CVE, victimization, i.e. firearm-related injury, has become the leading cause of death among children and adolescents in the US [13]. Other studies have also shown that CVE may lead to a number of mental health problems in children and youth, such as depression, posttraumatic stress, and anxiety, and impact their daily functioning, and that there may be a dose-response effect, where different degrees of CVE, namely witnessing and victimization, may impact mental health differently, with victimization having a stronger, and more damaging effect [14–16]. Moreover, although witnessing may have a less pronounced effect, it can still be a severe traumatic experience, that may have a very damaging impact on mental health through similar mechanisms.

Although Western culture with its ideals of thinness in relation to weight and shape has been thought to impact the risk and course of EDs, with anorexia nervosa being especially prevalent in White female populations [17], ED variants are present across diverse racial and ethnic groups and the prevalence of EDs is increasing in non-White individuals [18]. At the same time, while an overwhelming number of studies have reported an association between EDs and a wide range of traumatic experiences, such as child emotional abuse, emotional neglect and exposure to intimate partner violence [19, 20], childhood neglect [20, 21], and PTSD [11], most of these studies have been undertaken in high-income Western countries and are based on adult populations. In addition, previous research has indicated that the reported effects of different kinds of violence exposure on ED symptoms in adolescent populations may vary considerably and that there is unexplained heterogeneity in outcomes between different studies [22].

Hence, it remains unclear if the association between CVE and disordered eating would be similar for adolescents from different countries. Cross-cultural comparisons of the factors contributing to EDs are needed since cultural beliefs and attitudes may play a role in the development and treatment of EDs [18]. Moreover, as the rates of both exposure to traumatic events, such as CVE [12], and of EDs [23] vary substantially between different countries and by gender, there is a need to investigate if the degree of CVE is differentially associated with EDs depending on the country, socio-economic status (SES) and gender of the participants. Previous studies have reported that CVE is more common among ethnic minority adolescents residing in urban inner-city neighborhoods [24], and that more boys report CVE, including physical assault, than girls [25, 26]. Given that PTSD symptoms, and symptoms of depression and anxiety may moderate [10] the effects of violence exposure on disordered eating, there is also a need to adjust for such symptoms when analyzing these associations in order to evaluate whether an increasing degree of CVE might have a more direct effect on ED symptoms, independent of comorbidity.

To address these research questions, we used data from a large international study that collected information on CVE and ED symptoms from three culturally distinct samples, while also providing a gender perspective. Specifically, this study aimed to: (i) explore whether a greater degree of CVE was associated with increasing ED symptoms (both ED thoughts and compensatory behaviors) in adolescents from Belgium, Russia and the US; (ii), investigate whether the associations between CVE and ED symptoms were similar in different countries, and whether these associations were gender-specific; and (iii) evaluate if a greater degree of CVE (victimization) has a stronger effect on disordered eating, independent of the effect of comorbidity. We hypothesized that boys in the three countries would report higher rates of CVE than girls, whereas girls would have more ED symptoms than boys. We expected that an increasing degree of CVE would be associated with more ED symptoms and that this association may differ for boys and girls, but that the patterns of this association would nevertheless be generalizable across different countries. It was further hypothesized that the association between CVE and ED symptoms would be attenuated after including posttraumatic stress, depressive symptoms and anxiety symptoms in the model, but that the direct association between victimization and ED symptoms would nevertheless remain significant.

## Methods

### Procedure

The data used in this study were drawn from the Social and Health Assessment (SAHA) conducted in Belgium, Russia and the US. The primary aim of the SAHA was to determine the factors associated with adolescent health and well-being. The study sites were the following: Belgium (Antwerp with a population of 500,000), Russia (the city of Arkhangelsk with a population of 360,000), and the US (the city of New Haven, Connecticut, with a population of 125,000).

Details of the survey’s methodology have been previously published elsewhere [27–29]. In brief, in all three sites students aged 12–18 who were enrolled in the public school system were included. Students in Belgium and Russia were recruited from within classes that were randomly selected from schools that had themselves been randomly selected, whereas in the US all students in the specific age range who were in the school system were invited to participate. In each of the three countries, students completed the survey in their classrooms during a normal school day. The survey was administered by research project staff and by school system personnel in sessions lasting 45–60 min. All personnel were trained to administer the survey in a standardized way. Prior to the survey administration written informed consent was obtained from all participants, and both parents (for their children) and the children themselves had the right to decline to participate. Response rates for these surveys were high with only 1.4% of children in Belgium; 3.6% in Russia and < 1% in the US declining to participate.

### Participants

In total, 9751 students participated (2624 from Belgium, 2856 from Russia and 4271 from the US). Of these, 1030 students (10.6%) had missing data on at least one of the study variables, with most of this missing data (n = 419) concerning parental employment status. The age of the students ranged from 12 to 18 years (Mean age = 14.27 years, SD = 1.59; Mean age in Belgium = 14.68, in Russia = 14.91 and in the US = 13.59), and 50.7% of the total sample were girls (with females comprising 43.2% of the sample in Belgium, 57.6% in Russia, and 50.7% in the US). Before conducting multiple imputation (see the Statistical Analyses section), attrition analyses were performed. Those who had at least one missing data point were younger (t = 2.81; p = 0.005), were more frequently boys (χ² = 20.95; p < 0.001), and from the US (χ² = 56.05; p < 0.001), had a lower incidence of CVE (χ² = 18.75; p < 0.001), lower SES (t = 3.01; p = 0.003) and higher ratings on posttraumatic stress symptoms (t = 2.23; p = 0.026). Ethical approval for the study was obtained from the relevant ethical review boards in all three countries.

### Measures

#### Eating Disorder Symptoms

Symptoms were assessed using a shortened version of the Eating Disorder Diagnostic Scale [30] that inquiries about the presence of disordered eating behaviors and thoughts related to one’s shape and weight in the past 3 months. It consists of four statements on the occurrence of *ED thoughts*: “I worried a lot about how to stop gaining weight”, “I felt fat even when others told me I am too thin”, “I felt very upset about my overeating or weight gain” and “I ate large amounts of food even when I didn’t feel hungry”. Responses were scored using a three-point Likert scale: “not true” (scored 0), “somewhat true” (1), and “certainly true” (2). In the present study, the internal consistency of this symptom scale for the pooled sample was acceptable (Cronbach’s α = 0.76). In addition, two questions assessed the frequency of *compensatory behaviors* each week, which were used in relation to the ED symptoms in order to prevent weight gain: “vomiting or using laxatives (purging)” and “fasting (skipping at least 2 meals in a row) or engaging in excessive exercise”. These items were assessed using a five-point response scale ranging from “0 times” (scored 0) to “more than 10 times” (scored 4). Both subscales (i.e. on the occurrence of ED thoughts and the frequency of the compensatory behaviors) were used as continuous scales, with the potential scores ranging between 0 and 8 for each subscale.

#### Community Violence Exposure

Items assessing the witnessing of violence (seven items) and direct victimization (seven items) were derived from the Screening Survey of Exposure to Community Violence, developed by Richters and Martinez [31] (see Supplementary Table 1). The students were asked about “things that may happen to people in some neighborhoods”. Using a 5-point response scale format (ranging from “None” [scored 0] to “10 or more times” [4]) the students described whether they had witnessed violence or been victimized in the past year, which included such experiences as being beaten up or mugged, threatened with serious physical harm by someone, or seriously wounded in an incident of violence. Three groups were formed according to the reported types of exposure. Those who did not report any witnessing and victimization episodes were considered as the *non-exposed group* (coded 0). Those, who reported at least one episode of witnessing, but no episodes of victimization were considered as the *witnessing group* (coded 1). Finally, those, who reported at least one episode of victimization were considered the *victimization group* (coded 2).

#### Posttraumatic Stress

Symptoms were measured with the Child Post-Traumatic Stress Reaction Index (CPTS-RI), a 20-item scale, assessing posttraumatic stress in school-aged children and adolescents after exposure to trauma [32, 33]. The frequency of symptoms in the past month was assessed on a five-point Likert-type rating scale ranging from “never” (0) to “most of the time” (4), with a total score range from 0 to 80. The scale’s score is highly correlated with the DSM-based diagnosis of PTSD and has well-established clinical cut-offs. A score between 12 and 24 indicates mild posttraumatic stress, a score of 25–39 indicates moderate posttraumatic stress, 40–59 severe posttraumatic stress, and a score of 60 and above indicates very severe posttraumatic stress [33]. In this study, Cronbach’s alpha for the scale was 0.85.

#### Depressive Symptoms

Symptoms were measured using a modified version of the Center for Epidemiologic Studies-Depression Scale (CES-D) [34], which has shown excellent psychometric properties in adolescent populations [35]. The 10-item scale inquires about symptoms of depression (e.g. feeling lonely, feeling disliked and having problems sleeping) during the past month, with symptoms being assessed on a three-point scale: “not true” (0), “somewhat true” (1), “certainly true” (2). Scores were summed and could range between 0 and 20, with higher scores indicating an increased number of depressive symptoms. Cronbach’s alpha for the scale was 0.82.

#### Anxiety Symptoms

Symptoms were assessed using a 12-item scale [27] which targets worrisome and preoccupying thoughts or unpleasant feelings about the student him/herself or about external stimuli (e.g. feeling nervous when being called on in class, worrying about the future or worrying whether other people like him/her). The students reported these symptoms using a three-point scale: “not true” (0), “somewhat true” (1), “certainly true” (2). The total score could range between 0 and 24, with higher ratings indicating a higher number of anxiety symptoms. Cronbach’s alpha for the scale was 0.86.

#### Socioeconomic Status

SES was assessed by the students’ reports on the current employment status for each of their parents: unemployed (1), employed part-time (2), and employed full-time (3), with a possible score ranging up to 6 points for each family, where the score can be regarded as a proxy for the household’s financial condition (with higher scores representing higher SES). This factor has been described as being relevant in assessing SES in cross-cultural contexts [36].

### Statistical Analyses

Data were analyzed using SPSS, version 28. As indicated above by the results from the attrition analyses between those with and without missing data, Little’s MCAR test [37] that included all of the study variables was significant (χ2 = 55.06, DF = 14, p < 0.01), which indicates that the data were not missing completely at random. Given this, we used a multiple imputation procedure, deemed appropriate in previous studies [38, 39] and five sets of imputations were made using a standard fully conditional specification. In the descriptive tables we present the pooled data. One-way ANOVAs and post-hoc tests (Tukey HSD) were used for comparing ED symptom ratings (ED thoughts and compensatory behaviors), SES, posttraumatic stress symptoms, depressive symptoms and anxiety between the students from Belgium, Russia and the US, while independent sample t-tests were used to examine differences by gender, separately for each county. Chi-square tests were used for univariate comparisons of the prevalence of different degrees of CVE. Multivariate Analyses of Covariance (MANCOVA) were performed to assess the occurrence and frequency of ED symptoms in boys and girls, who reported different degrees of CVE (no exposure, witnessing, and victimization). The analysis was adjusted for age, country, gender and SES. As a separate analysis, we repeated the initial model, while adding PTSD symptoms, depressive symptoms and anxiety as co-variates in order to investigate if the association between CVE and ED symptoms remained significant. Lastly, we examined if country or gender moderated the association between the degree of CVE and ED symptoms using 3 (degree of CVE) × 3 (country), and 3 (degree of CVE) × 2 (gender) designs. Posttraumatic stress, depressive symptoms and anxiety were not included in these models. Two-tailed tests with p-values < 0.05 were considered statistically significant. We used the points of reference provided by Cohen [40] to define small (η2 = 0.01), medium (η2 = 0.06), and large (η2 = 0.14) effects.

## Results

### Group Comparisons

Table 1 shows the study variables by gender in Belgium, Russia and the US. Table 2 shows the prevalence of ED symptoms in Belgium, Russia and the US by the degree of CVE in boys and girls. No differences were found between the countries in the occurrence of ED thoughts (F(2, 9750) = 2.84; p = 0.061). However, the frequency of ED compensatory behaviors differed between the countries (F(2, 9750) = 44.77; p < 0.001), with US students having higher ratings than both Russian (p < 0.001) and Belgian (p = 0.004) students, and Belgian students having higher ratings than Russian students (p < 0.001). There was a significant difference between the countries in the reported degree of CVE (χ^2^ = 425.71; p < 0.001), with Belgian students reporting the highest rates of exposure to victimization and Russian students more often reporting no CVE. The countries differed in terms of posttraumatic stress (F(2, 9750) = 23.44; p < 0.001), depressive symptoms (F(2, 9750) = 94.87; p < 0.001) and anxiety (F(2, 9750) = 369.82; p < 0.001), with students from the US having higher posttraumatic stress ratings than those from Belgium and Russia (p < 0.001), students from Russia having higher ratings on depressive symptoms and anxiety than those from Belgium and the US (p < 0.001), and US students having higher ratings on anxiety than those from Belgium. The countries also differed in SES (F(2, 9750) = 118.44; p < 0.001), with US students having lower SES than both Belgian and Russian students. As shown in Table 1, across all countries, girls reported the occurrence of more ED thoughts than boys. Girls in Belgium and Russia also reported a higher frequency of ED compensatory behaviors than boys, while no gender differences were found in this regard among the US students. A higher proportion of girls than boys reported having witnessed violent events, whereas boys reported higher rates of victimization by violence than girls. As concerns posttraumatic stress, depression and anxiety in all countries, girls reported more symptoms than boys.

**Table 1:** Gender differences (t-test and Chi-square test) in the prevalence of eating problems, degree of CVE, posttraumatic stress symptoms, depressive symptoms and anxiety by country

	Belgium		Russia		US	
	Boys (n = 1483)	Girls (n = 1141)	Boys (n = 1208)	Girls (n = 1647)	Boys (n = 2095)	Girls (n = 2176)
ED thoughts Mean (SE)	1.25 (0.04)	2.50 (0.07)	0.99 (0.05)	2.43 (0.06)	1.37 (0.04)	2.43 (0.05)
Statistics	t = 15.03***		t = 19.01***		t = 14.88***	
Frequency of ED compensatory behaviors Mean (SE)	0.48 (0.03)	0.70 (0.04)	0.26 (0.03)	0.51 (0.0.02)	0.65 (0.03)	0.68 (0.03)
Statistics	t = 4.92***		t = 7.26***		t = 0.67	
Degree of CVE (%)						
No exposure	15.1	23.6	30.0	41.4	19.5	24.6
Witnessing	35.1	37.3	37.4	41.0	38.8	51.3
Victimization	49.8	38.9	32.2	17.7	41.8	24.0
Statistics	χ^2^ = 39.93***		χ^2^ = 90.07***		χ^2^ = 147.03***	
Posttraumatic stress Mean (SE)	17.76 (0.30)	22.66 (0.42)	17.84 (0.33)	21.58 (0.29)	19.52 (0.27)	23.85 (0.30)
Statistics	t = 9.53***		t = 8.56***		t = 10.67***	
Depressive symptoms Mean (SE)	3.67 (0.09)	7.76 (0.14)	5.08 (0.12)	6.65 (0.10)	3.76 (0.08)	5.69 (0.10)
Statistics	t = 12.64***		t = 10.07***		t = 14.67***	
Anxiety Mean (SE)	8.28 (0.13)	10.57 (0.17)	12.19 (0.16)	14.09 (0.13)	9.53 (013)	11.38 (0.12)
Statistics	t = 10.77***		t = 8.94***		t = 10.43***	

Note. ED: Eating disorder; CVE: community violence exposure; SE: standard error

**Table 2:** Descriptive statistics of eating disorder symptoms (M(SD)) in Belgium, Russia and the US by the degree of CVE in boys (B) and girls (G)

			Degree of CVE		
			Non-exposed	Witnessing	Victimization
Eating disorder thoughts	Belgium	B	0.87 (0.10)	1.18 (0.07)	1.42 (0.06)
		G	1.76 (0.13)	2.43 (0.11)	3.02 (0.11)
	Russia	B	0.72 (0.07)	0.88 (0.07)	1.37 (0.09)
		G	2.26 (0.09)	2.46 (0.09)	2.75 (0.14)
	US	B	1.13 (0.09)	1.22 (0.07)	1.63 (0.07)
		G	2.00 (0.10)	2.46 (0.08)	2.81 (0.11)
Eating disorder	Belgium	B	0.20 (0.05)	0.50 (0.05)	0.54 (0.05)
compensatory behaviors		G	0.37 (0.05)	0.64 (0.05)	0.96 (0.06)
	Russia	B	0.12 (0.03)	0.24 (0.04)	0.42 (0.06)
		G	0.40 (0.03)	0.55 (0.04)	0.70 (0.07)
	US	B	0.47 (0.05)	0.48 (0.03)	0.89 (0.05)
		G	0.41 (0.04)	0.64 (0.03)	1.02 (0.07)

The values presented are not adjusted for the list of covariates

CVE: Community violence exposure; M: mean; SD: standard deviation

### Predictors of ED Thoughts and Compensatory Behaviors

Table 2 shows the prevalence of ED symptoms across all countries by the degree of CVE in boys and girls, while Table 3 presents effect sizes for each dependent variable. The main effect for the type of exposure for the total sample was significant, with ED thoughts and the frequency of ED compensatory behaviors increasing together with an increasing degree of CVE (from no exposure to witnessing to direct victimization). In addition, the main effects for gender, country and SES were significant. More specifically, being female and having a lower SES were associated with increasing ED thoughts and more ED compensatory behaviors, while being Russian was associated with fewer ED symptoms whereas being from the US was associated with more ED compensatory behaviors. The model explained 9.5% of the variance in ED thoughts and 3.5% of ED compensatory behaviors.

**Table 3:** Main effects from the MANCOVA tests comparing ED thoughts and compensatory behaviors by the degree of CVE, country, and gender, while adjusting for age and socioeconomic status

	Summary statistics Wilks’ lambda, *F-value*(df), *η*^*2*^, *p-value*	ED thoughts *b, η2, p-value*,	ED compensatory behaviors *b, η2, p-value*
CVE	0.974, 65.15(4), 0.013, < 0.001		
Witnessing		0.28, 0.003, < 0.001	0.16, 0.003, < 0.001
Victimization		0.67, 0.014, < 0.001	0.43, 0.019, < 0.001
Country	0.994, 14.50(4), 0.003, < 0.001		
US (ref = Belgium)		0.09, 0.00, 0.118	0.11, 0.001, < 0.001
Russia (ref = Belgium)		-0.03, 0.000, 0.562	-0.11, 0.001, < 0.001
Russia (ref = US)		-0.12, 0.000, 0.031	-0.22, 0.001, < 0.001
Gender (ref = male)	0.912, 468.66(2), 0.088, < 0.001	1.30, 0.087, < 0.001	0.20, 0.007, < 0.001
Age	1.000, 1.30(2), 0.000, 0.285	0.02, 0.000, 0.126	0.00, 0.000, 0.746
SES	0.999, 5.85(2), 0.001, 0.001	-0.04, 0.001, 0.028	-0.03, 0.001, 0.008

Note CVE: community violence exposure; ED: eating disorder; SES: socioeconomic status; Ref: reference category

When adjusting for posttraumatic stress, depressive symptoms and anxiety, the associations were attenuated but remained significant both between the degree of CVE and ED thoughts (from no exposure to witnessing [b = 17; p = 0.001; η2 = 0.001] to victimization [b = 22; p < 0.001; η2 = 0.002]) and between the degree of CVE and ED compensatory behaviors (from no exposure to witnessing [b = 10; p < 0.001; η2 = 0.001] to victimization [b = 22; p < 0.001; η2 = 0.005]). The model explained 20.7% of the variance in ED thoughts and 8.7% of the variance in ED compensatory behaviors (see Supplementary Table 2).

### Interaction of Country and Gender on the Association between CVE and ED

A main effect of CVE x country (Wilks’ lambda = 0.997; F(8, 19,472) = 4.59, p < 0.001, η2 = 0.002), but not of CVE x gender (Wilks’ lambda = 0.999; F(4, 19,472) = 1.98, p = 0.098, η2 = 0.000) on ED symptoms was found. Specifically, this showed a smaller increase in ED thoughts and compensatory behaviors among Russian students who witnessed violence or experienced violent victimization.

## Discussion

The purpose of this cross-sectional study was to assess how the degree of CVE relates to ED symptoms (ED thoughts and compensatory behaviors) in Belgian, Russian and US adolescents, and whether the relation differed by country and gender. In all three countries, girls reported more ED symptoms, while boys reported being more frequently exposed to violent victimization than girls. An increasing degree of CVE (from no exposure to witnessing to victimization) was associated with more ED symptoms, with a small effect size. The association was similar for adolescents in all three countries, although the increase in ED symptoms was not as pronounced among Russian students. When adjusting for posttraumatic stress, depressive symptoms and anxiety, the association between CVE and ED symptoms remained significant.

In line with our hypothesis, girls reported more ED symptoms than boys. This gender difference was similar in all three countries, with gender having a medium size effect on ED symptoms. Victimization was more frequently reported by boys, whereas the witnessing of community violence was more common among girls. Our findings are in line with those from previous studies that have reported cross-cultural and cross-gender differences in the occurrence of EDs [23], where ED symptoms have been more associated historically with Western cultural ideals of thinness and more prevalent among females [17], as represented in our study by the students from Belgium and the US. Previous literature has also shown that boys more often report CVE victimization than girls [25, 26], and that CVE is more prevalent among adolescents of color from urban inner-city neighborhoods [24]. In our study, Belgian students had the highest rates of victimization. It is uncertain what underlies this difference, but hypothetically, it could reflect possible differences in reporting behaviors, and that the high prevalence of violence in Belgium might be related to the fact that the data originated from the largest city in the Flemish part, and a city with a large port, which has a larger immigrant population, and likely more deprivation in certain suburbs and higher rates of violence.

As hypothesized, a greater degree of CVE was associated with an increased prevalence of both ED thoughts and compensatory behaviors. In particular, those adolescents who reported having been victimized had more ED symptoms, which corroborates previous findings linking traumatic experiences with the occurrence of all types of EDs and their associated features [8, 19–22]. Importantly, the association between the degree of CVE and ED symptoms was similar across all three countries, strengthening the generalizability of our findings, albeit with a relatively smaller increase in ED symptoms among Russian students exposed to witnessing or victimization. These results thus underscore the burden of CVE across different contexts. At the same time, it should also be noted that the effect size of CVE on ED symptoms was small. Moreover, in opposition to our hypothesis, the interaction effect for the degree of CVE x gender on ED symptoms was not significant. Although there has been little research until now that has investigated gender-specific differences in the association between CVE and EDs, previous literature suggests that females are at an increased risk of developing PTSD [41, 42] and internalizing mental health problems [43] when experiencing CVE, as compared to their male counterparts, contrasting our finding of a lack of gender differences when ED symptoms are used as an outcome.

The association between victimization and EDs was attenuated when also including symptoms of posttraumatic stress, depression and anxiety in the model, indicating that internalizing symptoms may partly account for the association between a traumatic exposure and ED symptoms, which corresponds with previous research findings [10]. It has also been suggested that PTSD and EDs share common risk factors, including exposure to traumatic events [9]. However, our findings also indicate a specific and unique effect of the degree of CVE on ED symptoms, which is independent of comorbid symptoms, including posttraumatic stress. It has been suggested that the patterns of emotional response to trauma, such as avoidance, suppression, and dissociation might be important for EDs [44], and these patterns are also closely linked with PTSD. It has also been hypothesized that ED behaviors may be used as coping strategies to manage trauma-related distorted beliefs, such as self-criticism, low self-worth, or self-punishment [10]. Further, stressful life events have been recurrently associated with disordered eating behaviors [45], as well as with experiencing a relapse in EDs [46]. Traumatic events are experienced by an individual as phenomena that extend beyond his/her immediate capacity to manage the situation and as something that negatively affects well-being [47], a description that might also be appropriate for some individuals exposed to community violence. In this context it has been suggested that disordered eating behaviors, such as restricting the amount of food eaten and binge eating may be used as strategies that are temporarily effective in managing emotional responses induced by stressors in life [45], including witnessing of and victimization by community violence. For instance, it has been discussed that binge eating and purging might serve as strategies to numb emotions and reduce emotional arousal, whereas dietary restriction may act as a regulator of emotions by reducing ambiguity and providing simplified rules for daily functioning [10].

Our study has several strengths, including the use of a large sample of community-based adolescents from three culturally different locations. Nonetheless, there are several limitations that should be mentioned. We relied entirely on self-reported ratings, which may result in different forms of bias, such as recall bias and social desirability bias [48]. Even though adolescent mental health problems, specifically internalizing symptoms, are best measured with self-ratings [49], using data from other information sources, such as parents, or data collected through a structured diagnostic interview, would have improved confidence in our findings. Further, the use of other data such as that relating to body mass index, which was unfortunately not available, would have provided important additional information with regard to ED symptom severity. Similarly, we also lacked information on other factors that might have affected the investigated association, such as puberty status, social attitudes and norms.

Overall, the effect sizes were small, except for a moderate effect of gender on ED symptoms. According to the developmental psychopathology framework, a wide range of risk and protective factors (biological, psychological, social-contextual, and the interactions among these) need to be considered across development given the complexity of normal and abnormal development [50]. Inclusion of other factors that may contribute to ED symptoms such as participation in weight-oriented sports, heritability [6], or other traumatic experiences such as neglect, emotional and sexual abuse [6, 8], could have strengthened our findings. In addition, previous literature suggests that some factors from the immediate environment of the students, such as parental involvement and teacher support, may have a protective role against traumatic experiences [51]. Further, the timing and chronicity of the CVE may be of importance, since traumatic exposures that span over several developmental periods may be more detrimental for the individual, and the outcomes might also be more diverse, including other psychiatric symptoms [52]. As our study used cross-sectional data, it was not possible to determine the temporal nature of the associations, or establish causality. Finally, it should be noted that our study also lacked data on any interventions targeting mental health problems that the participants may have received, which might have impacted on the outcomes. Future studies should employ longitudinal cross-cultural designs, and also include possible protective factors, in order to determine whether CVE predicts ED symptoms over time, while also looking at the gender-specific and cross-cultural patterns in these associations.

## Summary

This study found that CVE, and especially victimization was associated with ED symptoms, and that the association was comparable for boys and girls in three culturally different samples. This finding raises the issue of the potential need for interventions targeting factors contributing to ED symptoms, such as trauma in general and CVE in particular. Further identification of traumatic events in the etiology of EDs may facilitate prevention efforts with regard to early adverse influences on the developing child, and enable psychotherapy targeting traumatic stress. This is of particular interest given that CVE represents one of the most common adverse childhood experiences [12]. Future research would benefit from using longitudinal designs, adjusting for co-occurring symptoms such as posttraumatic stress, and exploring the potential mechanisms driving the associations in more detail.

## Electronic Supplementary Material

Below is the link to the electronic supplementary material.

Supplementary Material 1

Supplementary Material 2

## Data Availability

The datasets used in this article are not readily available because the data cannot be shared publicly due to the initial decision of the local ethical committees, as well as the restrictions included in the informed consent statement (where it was stated that the data would only be used by the research group and would not be transferred elsewhere). Requests to access the datasets should be directed to vladislav.ruchkin@neuro.uu.se.
