# Supplementary material for: Community Violence Exposure and Eating Disorder Symptoms among Belgian, Russian and US Adolescents: Cross-Country and Gender Perspectives
Source: Child Psychiatry Hum Dev. 2023 Aug 22;56(3):595–604. doi: 10.1007/s10578-023-01590-1 (PMC12095450; doi:10.1007/s10578-023-01590-1)
Supplement: Supplementary file 1 — Supplementary Material 1 [file 10578_2023_1590_MOESM1_ESM.docx]

**Supplementary Table 1**. The items used for assessing witnessing of and victimization by community violence

| **In the past year, I have seen…** |
| --- |
| Someone else getting beaten up or mugged |
| Someone else get threatened with serious physical harm |
| Someone else get shot or shot at with a gun |
| Someone else being attacked or stabbed with a knife |
| Someone else being chased by gangs or individuals |
| A seriously wounded person after an incident of violence |
| Someone else get threatened or harmed because of race or ethnicity |
| **In the past year, I have been…** |
| Beaten up or mugged |
| Threatened with serious physical harm by someone |
| Shot or shot at with a gun |
| Attacked or stabbed with a knife |
| Chased by gangs or individuals |
| Seriously wounded in an incident of violence |
| Someone else get threatened or harmed because of race or ethnicity |
