# Supplementary material for: Community Violence Exposure and Eating Disorder Symptoms among Belgian, Russian and US Adolescents: Cross-Country and Gender Perspectives
Source: Child Psychiatry Hum Dev. 2023 Aug 22;56(3):595–604. doi: 10.1007/s10578-023-01590-1 (PMC12095450; doi:10.1007/s10578-023-01590-1)
Supplement: Supplementary file 2 — Supplementary Material 2 [file 10578_2023_1590_MOESM2_ESM.docx]

**Supplementary Table 2** Main effects from the MANCOVA tests comparing ED thoughts and compensatory behaviours by the degree of CVE, country, and gender, while adjusting for age, socioeconomic status, posttraumatic stress, depressive symptoms and anxiety.

|  | Summary statistics  Wilks’ lambda, *F-value*(df), *η^2^, p-value* | ED thoughts  *b, η2, p-value* | ED compensatory behaviors  *b, η2, p-value* |
| --- | --- | --- | --- |
| CVE  Witnessing  Victimization | 0.994, 14.17(4), 0.003, <0.001 | 0.17, 0.001, 0.001  0.22, 0.002, <0.001 | 010, 0.001, <0.001  0.22, 0.005, <0.001 |
| Country  US (ref = Belgium)  Russia (ref = Belgium)  Russia (ref = US) | 0.988, 31.11(4), 0.006, <0.001 | -0.05, 0.000, 0.305  -0.46 0.007, <0.001,  -0.40, 0.006, <0.001 | 0.06, 0.000, 0.027  -0.19, 0.004, <0.001  -0.25, 0.007, <0.001 |
| Gender (ref=male) | 0.954, 232.54(2), 0.046, <0.001 | 0.88, 0.044, <0.001 | 0.49, 0.000, 0.038 |
| Age | 1.000, 1.44(2), 0.000, 0.240 | 0.01, 0.000, 0.495 | -0.09, 0.000, 0.229 |
| SES | 1.000, 2.33(2), 0.000, 0.142 | -0.01, 0.000, 0.449 | -0.02, 0.000, 0.075 |
| Posttraumatic stress | 0.991, 46.49(2), 0.009, <0.001 | 0.02, 0.005, <0.001 | 0.01, 0.007, <0.001 |
| Depressive symptoms | 0.971, 144.76(2), 0.029, <0.001 | 0.09, 0.021, <0.001 | 0.05, 0.017 <0.001 |
| Anxiety | 0.966, 168.24(2), 0.034, <0.001 | 0.07, 0.030, <0.001 | -0.02, 0.000, 0.465 |

Note CVE: community violence exposure; ED: eating disorder; SES: socioeconomic status; Ref: reference category
